# Characterization of Umbilical Cord Mesenchymal Stromal Cells Overexpressing Klotho and In Vitro Anti-Aging Efficacy of Their Derived Extracellular Vesicles

**DOI:** 10.3390/ijms27188326

**Published:** 2026-09-19

**Authors:** Anyuan Zhang, Shan Wang, Jing Zhang, Bianlei Yang, Xiaorong Su, Wenying Deng, Hongxiang Wang, Zhichao Chen, Qiubai Li

**Affiliations:** 1 Department of Rheumatology and Immunology, Union Hospital, Tongji Medical College, Huazhong University of Science and Technology, Wuhan 430022, China; 2 Department of Hematology, Union Hospital, Tongji Medical College, Huazhong University of Science and Technology, Wuhan 430022, China; 3 Department of Hematology, The Central Hospital of Wuhan, Tongji Medical College, Huazhong University of Science and Technology, Wuhan 430014, China

**Keywords:** mesenchymal stromal cells, extracellular vesicles, klotho, cellular senescence, osteogenic differentiation

## Abstract

Age-related degeneration remains a premier biomedical challenge, frequently underpinned by cellular senescence and stem cell dysfunction. Accumulating evidence suggests that umbilical cord mesenchymal stromal cell (UC-MSC)-derived extracellular vesicles (UC-EVs) and the longevity protein Klotho hold great promise as anti-aging biotherapeutics. Here, we strategically engineered UC-MSCs to overexpress Klotho and generated functional Klotho-enriched UC-EVs (KL-EVs). Our data demonstrated that genetic modification endowed UC-MSCs with prominent osteogenic differentiation potency and angiogenesis efficacy. More importantly, KL-EVs profoundly rescued multifaceted senescent phenotypes in both adult bone marrow mesenchymal stem cells (BMSCs) and human renal tubular epithelial cells (HK-2) in vitro. In particular, KL-EVs rescued the age-related differentiation bias of adult BMSCs by robustly prompting their osteogenic differentiation capacity. Taken together, we developed a novel type of UC-MSCs and isolated their EVs enriched with Klotho protein. This strategy integrated and enhanced the anti-aging effects of both entities in vitro, offering a new perspective for counteracting cellular senescence and alleviating age-associated degenerative alterations.

## 1. Introduction

Umbilical cord mesenchymal stromal cells (UC-MSCs), originating from the mesoderm, represent the most primitive population of adult stem cells harvested from human tissue. Renowned for their robust self-renewal capacity and multipotent differentiation potential, UC-MSCs are heralded as the quintessential “seed cells” in regenerative medicine [1,2]. Current consensus indicates that the therapeutic benefits of UC-MSCs largely depend on the extracellular vesicles (EVs) they release in a paracrine manner [3,4]. Growing evidence, alongside our earlier work, has confirmed that UC-MSC-derived EVs (UC-EVs) deliver a wide assortment of bioactive components to exert anti-inflammatory, pro-angiogenetic and antioxidative effects, hence efficiently ameliorating degenerative changes in aged bone and kidney [5,6]. Based on this, engineering of UC-EVs to further amplify their intrinsic therapeutic properties has been brought into the limelight, providing substantial preclinical evidence for clinical translation of innovative cell-free gerotherapeutics [7,8,9].

A major target for molecular anti-aging interventions is Klotho, a longevity gene first identified by Kuro et al., whose deficiency spawns a premature aging syndrome in mice, including shortened lifespan, atherosclerosis, skin atrophy, and osteoporosis [10]. Full-length Klotho operates as a transmembrane protein featuring an intracellular domain, a membrane anchor, and an extracellular domain with two functional parts, KL1 and KL2 [11]. Importantly, the expression level and activity of Klotho decline progressively during natural aging and across various age-related pathologies [12,13,14]. Numerous studies have demonstrated that replenishing Klotho by exogenous genetic delivery or recombinant protein administration held therapeutic efficacy against multiple degenerative pathologies [15,16,17]. Although promising, significant translational barriers remain. Recombinant Klotho protein production poses significant technical challenges, and its poor in vivo stability necessitates frequent, high-dose injections to maintain therapeutic levels [18]. Conversely, viral vector-based gene therapy carries inevitable genotoxic risks [19]. These limitations suggested that developing a biocompatible and stable delivery system that preserves Klotho bioactivity while ensuring sustained therapeutic delivery should be put high on the agenda. Of note, available evidence suggests that EVs may provide a biologically relevant context for Klotho delivery and expand the therapeutic potential of Klotho while facilitating the preservation and realization of its biological activity [20,21,22].

For this end, we established a targeted engineering strategy by leveraging UC-EVs as a natural, highly biocompatible delivery platform to increase Klotho abundance in the resulting EV preparations, thereby exploiting their potential. By genetically modifying UC-MSCs to overexpress Klotho, we successfully harvested EVs enriched with functional Klotho (KL-EVs). In this study, we explored the rejuvenating effects of these engineered EVs on two key target cells: adult bone marrow mesenchymal stem cells (BMSCs) and human renal tubular epithelial cells (HK-2). Our in vitro findings revealed that KL-EVs could lower cellular senescence markers in both cell types and markedly strengthen osteogenesis of BMSCs. Altogether, our work showed that engineering UC-EVs with Klotho would provide a potent cell-free strategy to combat cellular senescence and restore lineage plasticity, offering a promising therapeutic avenue for age-related tissue degeneration.

## 2. Results

### 2.1. Construction and Characterization of UC-MSCs Overexpressing Klotho

**Figure 1:**
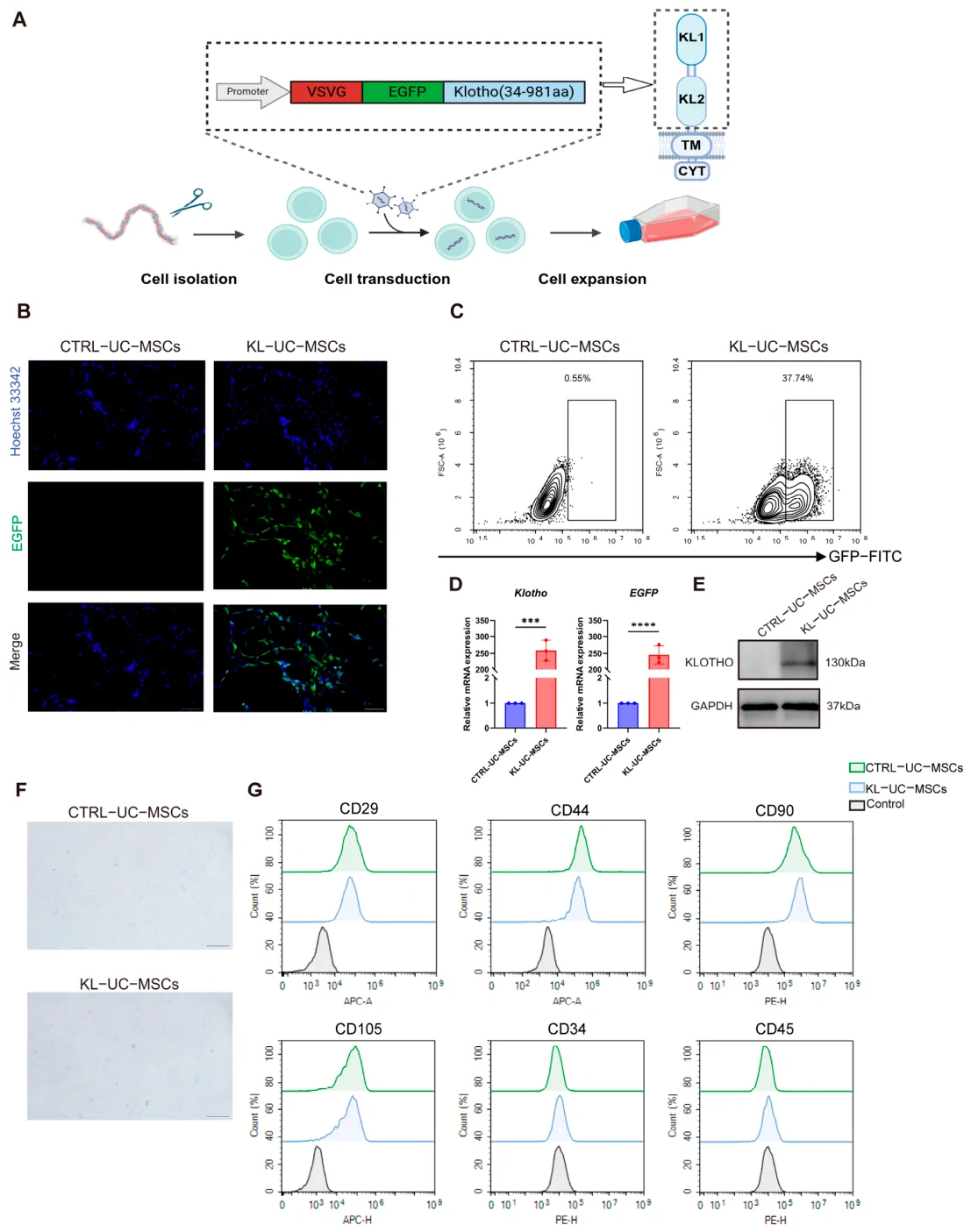
**Preparation of Klotho-overexpressed UC-MSCs.** (**A**) Schematic illustration of Klotho overexpressing lentivirus construction for transduction. (**B**) Representative fluorescence images showing GFP expression in KL-UC-MSCs (P5) and CTRL-UC-MSCs (P5). Scale bar, 200 μm. (**C**) Determination of the percentage of EGFP-positive cells in nonengineered and engineered UC-MSCs by flow cytometry. (**D**) mRNA expression of *Klotho* and *EGFP* in KL-UC-MSCs vs. CTRL-UC-MSCs assessed by RT-qPCR (*n* = 3). (**E**) Klotho protein expression levels of the two types of UC-MSCs detected by Western blotting. (**F**) Representative micrographs showing morphological features of both types of UC-MSCs (P5). Scale bar, 200 μm. (**G**) Immunophenotypic analysis of the surface markers CD29, CD44, CD90, CD105, CD34, and CD45 in CTRL-UC-MSCs and KL-UC-MSCs by flow cytometry. Data are presented as means ± SD. qPCR data were normalized to *GAPDH.* Panel (**D**) was analyzed by unpaired *t*-test (*** *p* < 0.001, **** *p* < 0.0001).

To enhance Klotho expression in UC-MSCs and promote its enrichment in their derived EVs, we employed vesicular stomatitis virus glycoprotein (VSVG) as a scaffold protein. Notably, to circumvent potential functional inactivation arising from the structural complexity of full-length Klotho, we strategically engineered a truncated version of Klotho (34-981, containing KL1 and KL2) fused to the VSVG scaffold (Figure 1A).

UC-MSCs were transduced with lentiviral vectors encoding the VSVG-Klotho ectodomain fusion protein (tagged with EGFP) or a negative control to generate KL-UC-MSCs and CTRL-UC-MSCs, respectively, and green fluorescence was observed (Figure 1B) in the transduced MSCs. Robust and stable transgene expression was confirmed by RT-qPCR, Western blotting and flow cytometry (Figure 1C–E), indicating successful UC-MSC engineering.

Subsequently, morphological and immunophenotypic analyses were performed on CTRL-UC-MSCs and KL-UC-MSCs. We observed that both cell populations exhibited a typical flat, elongated fusiform morphology and were arranged in swirl (Figure 1F). Flow cytometry showed that in line with CTRL-UC-MSCs, KL-UC-MSCs expressed the canonical MSC surface markers CD29, CD44, CD90, and CD105, and did not express the hematopoietic markers CD34 and CD45 (Figure 1G), suggesting that the engineered UC-MSCs retained their original basic immunophenotypic characteristics.

### 2.2. KL-UC-MSCs Exhibited Enhanced Osteogenic and Angiogenic Potential

**Figure 2:**
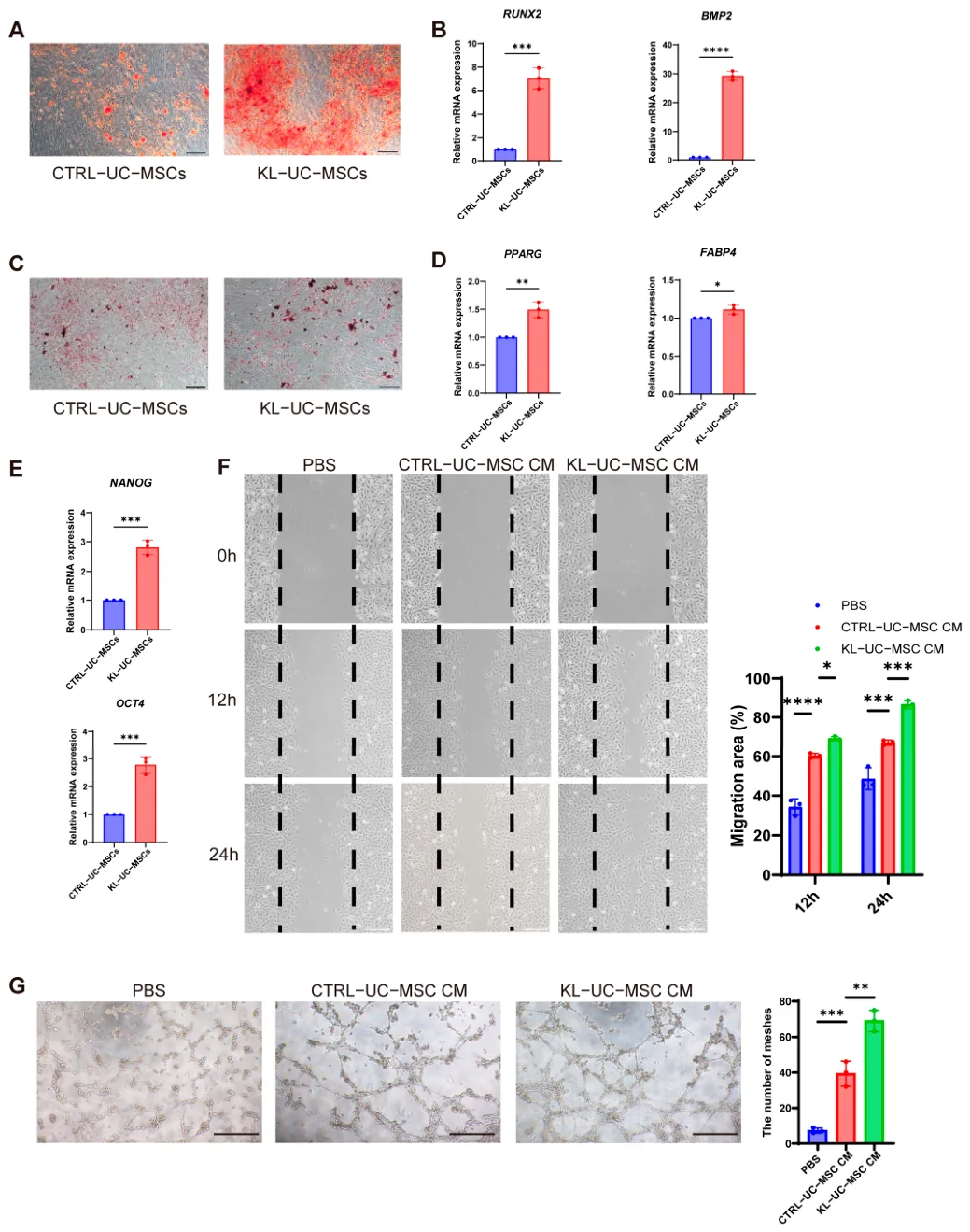
**Multidirectional differentiation and pro-angiogenesis capacity of KL-UC-MSCs.** (**A**) Representative images of Alizarin red S staining of UC-MSCs after osteogenic differentiation for 14 days (*n* = 3). Scale bars, 200 μm. (**B**) mRNA expression of osteogenic differentiation marker genes *RUNX2* and *BMP2* in UC-MSCs assayed by RT-qPCR (*n* = 3). (**C**) Representative images of oil red O staining of lipid accumulation in UC-MSCs after adipogenic differentiation for 14 days (*n* = 3). Scale bars, 200 μm. (**D**) mRNA expression of adipogenic differentiation marker genes *PPARG* and *FABP4* in UC-MSCs assayed by RT-qPCR (*n* = 3). (**E**) mRNA expression of stemness-related genes *NANOG* and *OCT4* assessed by RT-qPCR (*n* = 3). (**F**) Scratch wound assay to assess the promigratory effect of UC-MSCs and quantitative analysis of the wound recovery rate (*n* = 3). Black dashed lines indicate the edges of cell migration. Scale bars, 200 μm. (**G**) Tube formation assay to examine the pro-angiogenic effect of UC-MSCs and quantitative analysis of the number of meshes (*n* = 3). Scale bars, 500 μm. Data are presented as means ± SD. qPCR data were normalized to *GAPDH.* Panels (**B**,**D**,**E**) were analyzed by unpaired *t*-test. Panel (**F**) was analyzed by two-way ANOVA. Panel (**G**) was analyzed by one-way ANOVA (* *p* < 0.05, ** *p* < 0.01, *** *p* < 0.001, and **** *p* < 0.0001).

To compare the multilineage differentiation potential of CTRL-UC-MSCs and KL-UC-MSCs, we conducted in vitro osteogenic and adipogenic induction assays. Alizarin red staining revealed scattered, punctate red calcium nodules in CTRL-UC-MSCs after induction, whereas KL-UC-MSCs displayed prominent, sheet-like mineralized nodules (Figure 2A). Consistently, qPCR analysis of the induced cells further confirmed significant upregulation of osteogenesis-related genes *RUNX2* and *BMP2* in KL-UC-MSCs (Figure 2B). On the other hand, after adipogenic induction, lipid accumulation was observed within both CTRL-UC-MSCs and KL-UC-MSCs using the lipophilic dye Oil Red O (Figure 2C). qPCR analysis revealed a trend toward upregulated expression of adipogenic-related genes *PPARG* and *FABP4* in KL-UC-MSCs (Figure 2D). Interestingly, it was also found that the expression levels of stemness-associated genes *NANOG* and *OCT4* in KL-UC-MSCs were much higher (Figure 2E).

Given that pro-angiogenesis constitutes an indispensable basis for the tissue repair function of UC-MSCs, we next collected conditioned media (CM) from CTRL-UC-MSCs and KL-UC-MSCs to decipher the differences in terms of pro-angiogenesis. To begin, the scratch wound healing assay was performed to simulate the in vivo wound healing process, assessing the effects of the two types of UC-MSC CM on the migration ability of human umbilical vein endothelial cells (HUVECs). Results showed that, compared with the PBS-treated control group, both CTRL-UC-MSC and KL-UC-MSC CM reduced wound area. It is worth noting that the KL-UC-MSC CM group exhibited higher endothelial cell migration rates and more pronounced wound closure (Figure 2F). Furthermore, the tube formation assay revealed that HUVECs cultured with either CTRL-UC-MSC or KL-UC-MSC CM formed complete capillary-like structures, with the KL-UC-MSC CM group showing a significantly greater number of networked tubes (Figure 2G). These findings demonstrated that elevated intracellular Klotho expression maintained stemness of UC-MSCs and contributed to superior osteogenic differentiation capacity and pro-angiogenic function.

### 2.3. Klotho-Enriched EVs Were Isolated and Characterized

**Figure 3:**
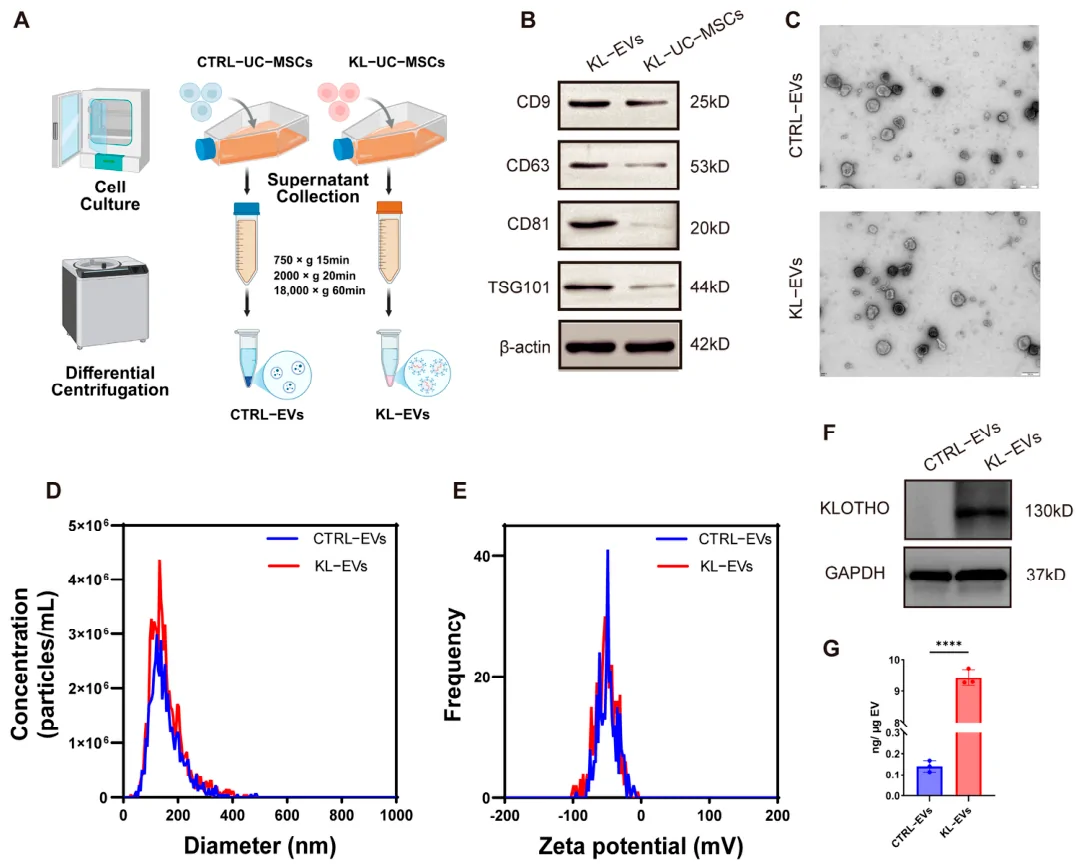
**Characterization of UC-EVs enriched with Klotho.** (**A**) Schematic illustration of the isolation protocol for CTRL-EVs and KL-EVs. (**B**) KL-EVs were positive for CD9, CD63, CD81, and TSG101 by using Western blotting. (**C**) Transmission electron microscopy images of EVs from two types of UC-MSCs. Scale bar, 200 nm. (**D**) The particle size and concentration of CTRL-EVs and KL-EVs were measured by nanoparticle tracking analysis. (**E**) The Zeta potential (mV) of CTRL-EVs and KL-EVs were measured by ZetaView. (**F**) Klotho protein levels in CTRL-EVs and KL-EVs were determined using Western blotting. (**G**) The levels of Klotho in EV lysate were measured by ELISA (*n* = 3). Panel (**G**) was analyzed by unpaired *t*-test (**** *p* < 0.0001).

It is universally acknowledged that EVs can convey various bioactive components, reflecting the state of the parent cells and reshaping phenotypes of the receptor cells [23]. To ascertain the alterations of Klotho abundance in EVs derived from engineered UC-MSCs and delve into their anti-aging potential, we collected culture supernatants from CTRL-UC-MSCs and KL-UC-MSCs and isolated their EVs via differential centrifugation (Figure 3A). Hereinafter, we refer to the EVs derived from KL-UC-MSCs as KL-EVs and those from CTRL-UC-MSCs as CTRL-EVs. It was found that KL-EVs expressed the canonical EV markers CD9, CD63, CD81, and TSG101 (Figure 3B), and exhibited spheroidal morphology with intact membrane structures comparable to CTRL-EVs under transmission electron microscopy, with most vesicles measuring less than 200 nm in diameter (Figure 3C). In agreement with this, nanoparticle tracking analysis was conducted to determine the diameter and concentration of both types of EVs, and the results indicated that the average size of EVs in both groups ranged from 130 to 150 nm (Figure 3D). To further elucidate the properties of EVs, we measured the Zeta potential of each EV group and the analysis revealed that the surfaces of CTRL-EVs and KL-EVs displayed a similar negative charge (Figure 3E). Strikingly, Western blotting demonstrated barely detectable Klotho expression in CTRL-EVs, whereas Klotho abundance was markedly increased in KL-EVs (Figure 3F). The levels of Klotho in both EV preparations were further quantified by ELISA, revealing over 9.0 ng of Klotho per μg KL-EV protein. These results confirmed the enrichment of Klotho protein in KL-EVs, laying the groundwork for the subsequent research.

### 2.4. KL-EVs Rejuvenated Adult BMSCs and Favorably Promoted Osteogenesis

BMSCs are capable of differentiating into various cell types, including osteoblasts and adipocytes. With aging, BMSCs exhibit significant functional decline, characterized by cell cycle arrest, reduced osteogenesis and enhanced adipogenesis [24,25]. As such, we delved into the efficacy of KL-EVs to ameliorate age-associated alterations of adult BMSCs.

**Figure 4:**
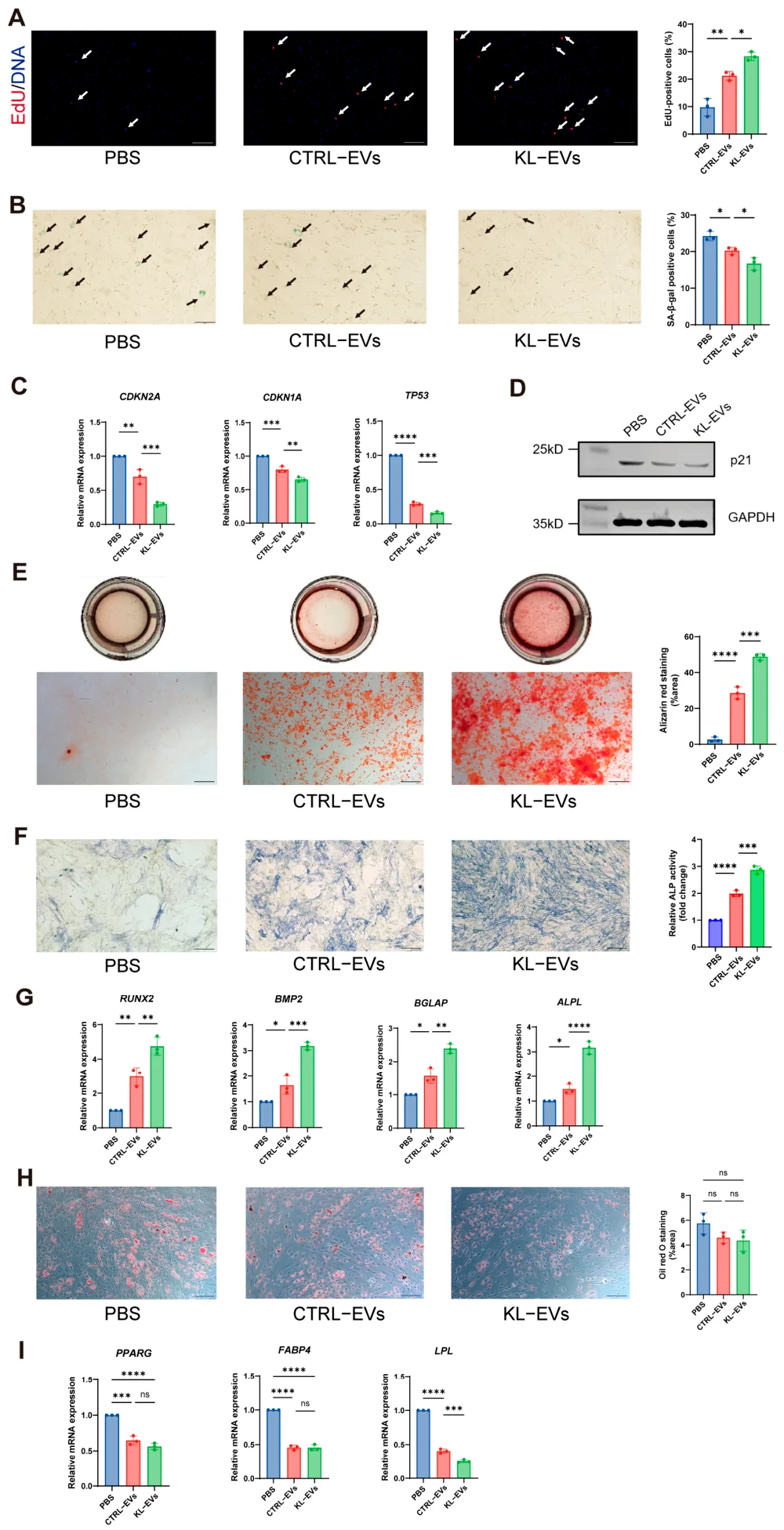
**KL-EVs improved the senescent phenotypes of adult BMSCs and significantly promoted osteogenic differentiation.** (**A**) Representative EdU labeling of cellular proliferation and quantification of BMSCs treated with PBS, CTRL-EVs or KL-EVs. Arrows indicate EdU-positive cells. (*n* = 3). Scale bars, 200 μm. (**B**) Representative senescence-associated β-galactosidase staining and quantification of BMSCs in different treatment groups. Arrows indicate SA-β-gal-positive cells. (*n* = 3). Scale bars, 200 μm. (**C**) mRNA expression of senescence-related marker genes *CDKN2A*, *CDKN1A*, and *TP53* in BMSCs assayed by RT-qPCR (*n* = 3). (**D**) Protein expression level of p21 was determined by Western blotting. (**E**) Representative images of Alizarin red S staining of BMSCs after EV treatment and osteogenic differentiation (*n* = 3). Scale bars, 200 μm. (**F**) Representative images and quantification of alkaline phosphatase staining of BMSCs in different treatment groups (*n* = 3). Scale bars, 200 μm. (**G**) mRNA expression of osteogenic differentiation marker genes *RUNX2*, *BMP2*, *BGLAP* and *ALPL* in BMSCs assayed by RT-qPCR (*n* = 3). (**H**) Representative images of oil red O staining of lipid accumulation in BMSCs after EV treatment and adipogenic differentiation (*n* = 3). Scale bars, 200 μm. (**I**) mRNA expression of adipogenic differentiation marker genes *PPARG*, *FABP4*, and *LPL* in BMSCs assayed by RT-qPCR (*n* = 3). Data are presented as means ± SD. qPCR data were normalized to *GAPDH.* All panels were analyzed by one-way ANOVA. (ns, nonsignificant, * *p* < 0.05, ** *p* < 0.01, *** *p* < 0.001, and **** *p* < 0.0001).

Initially, EdU incorporation assay demonstrated that while both EVs rescued the impaired proliferative vigor of adult BMSCs, KL-EV treatment achieved a significantly higher proportion of EdU-positive cells (Figure 4A). Moreover, SA-β-gal staining revealed that KL-EVs showed a superior capability to eradicate the senescent phenotype (Figure 4B). Consistently, CTRL-EVs and KL-EVs robustly downregulated the transcription of canonical senescent biomarkers, including *CDKN2A* (encoding p16), *CDKN1A* (encoding p21), and *TP53* (encoding p53) (Figure 4C), and this geroprotective transcriptional gene repression was further validated in the protein expression level of p21 in the KL-EV group (Figure 4D).

Next, we assessed the effects of CTRL-EVs and KL-EVs on the multidirectional differentiation potential of adult BMSCs. Alizarin red S staining provided striking macroscopic evidence of calcium mineral deposition. While the PBS control group exhibited virtually no red precipitates and the CTRL-EV group formed only sparse calcium deposits restricted to the well peripheries, KL-EV treatment robustly triggered extensive, confluent red nodular aggregates. Microscopic validation perfectly mirrored these gross examinations, confirming that KL-EVs uniquely induced the configuration of widespread, highly dense, sheet-like calcified matrix nodules, distinctly outstripping the scattered, point-like clusters observed in the CTRL-EV group (Figure 4E). In addition, alkaline phosphatase (ALP) enzymatic activity showed minimal baseline expression in the negative control. Although both EVs were demonstrated to augment intracellular ALP levels, the blue chromogenic deposition was much more pronounced, uniform, and intensive in BMSCs treated with KL-EVs (Figure 4F). To provide a definitive molecular resolution for these morphological transformations, we collected post-induction cell lysates to quantify the transcriptional activation of the core osteogenic genes via qPCR. As expected, both CTRL-EVs and KL-EVs upregulated the mRNA expression levels of pivotal osteoblast-lineage determinants, including *RUNX2*, *BGLAP* (encoding OCN), and *ALPL* (encoding ALP). Notably, KL-EVs possessed a drastically superior capacity to drive the transcription of these specialized genetic markers compared to their native counterparts (Figure 4G). In contrast to the robust osteogenic potential, the regulatory impact of these EVs on the reciprocal adipogenic lineage was subtle. Oil Red O staining showed that while both CTRL-EVs and KL-EVs partially restricted intracellular lipid droplet accumulation in adult BMSCs, the morphological reduction in adiposity did not achieve statistical significance (Figure 4H). Likewise, qPCR analysis demonstrated that both vesicles successfully downregulated the transcription of pivotal adipogenic marker genes, but basically no pronounced statistical variance was established between them (Figure 4I). Collectively, these findings showed that KL-EVs partially rejuvenated adult BMSCs, with the most prominent effect being the robust restoration of their dampened osteogenic differentiation capacity.

### 2.5. KL-EVs Performed Comprehensive Geroprotective Regulation of Senescent HK-2 Cells

**Figure 5:**
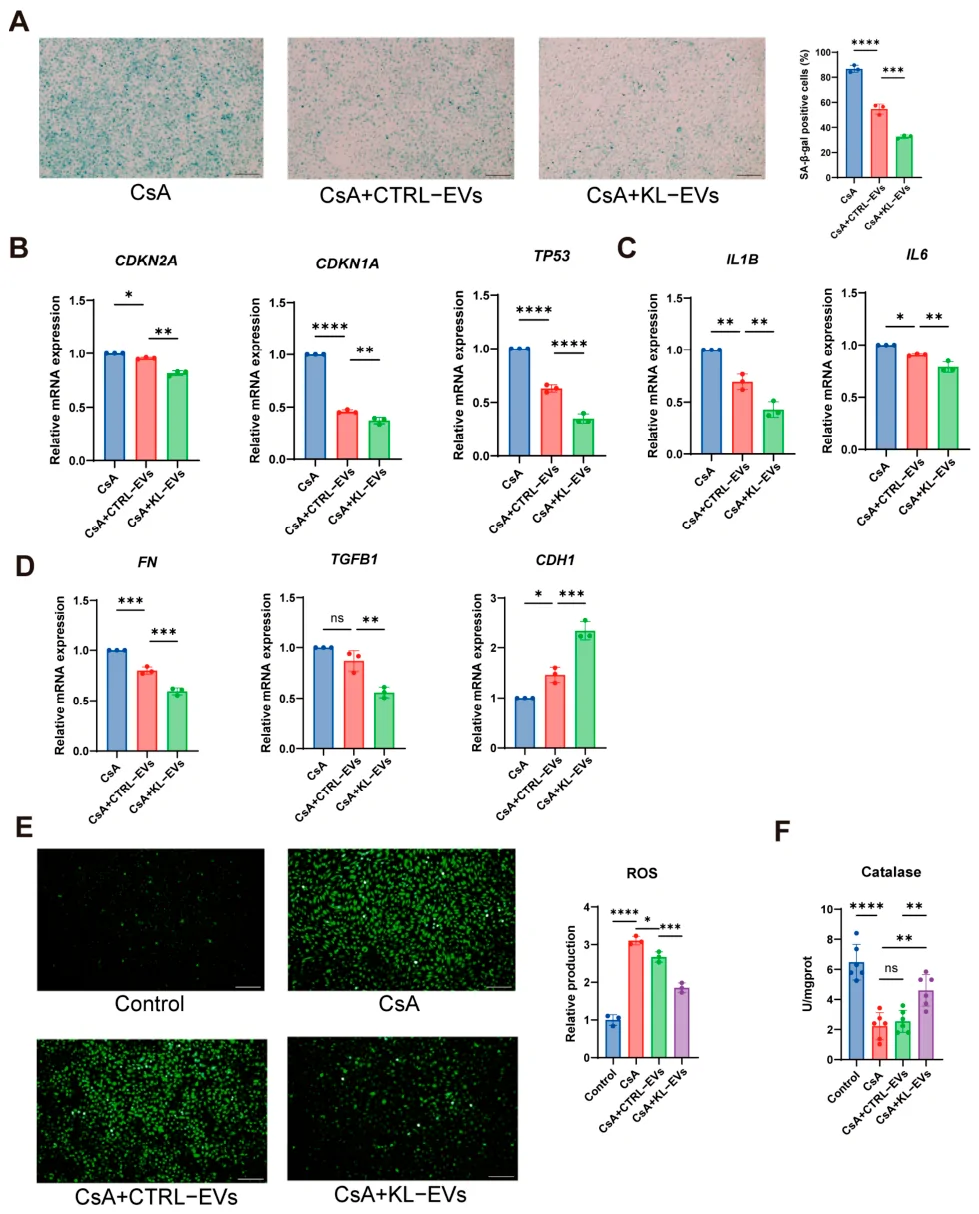
**KL-EVs effectively reverse multiple aging characteristics of HK-2 cells.** (**A**) Representative senescence-associated β-galactosidase staining and quantification of senescent HK-2 cells in different treatment groups. (*n* = 3). Scale bars, 500 μm. (**B**–**D**) mRNA expression of cellular senescence and epithelial–mesenchymal transition-associated marker genes in HK-2 cells were assessed by RT-qPCR (*n* = 3). (**E**) Detection of ROS levels in senescent HK-2 cells and bar graph showing the relative ROS production (*n* = 3). Scale bars, 200 μm. (**F**) Quantitation of catalase (CAT) levels in senescent HK-2 cells in different treatment groups (*n* = 6). Data are presented as means ± SD. qPCR data were normalized to *ACTB.* All panels were analyzed by one-way ANOVA. (ns, nonsignificant, * *p* < 0.05, ** *p* < 0.01, *** *p* < 0.001, and **** *p* < 0.0001).

In light of the inextricable link between Klotho and the senescence of renal tubular epithelial cells [26], we further investigated the therapeutic effects of KL-EVs on the age-related changes in senescent HK-2 cells. SA-β-gal staining revealed that since both CTRL-EVs and KL-EVs partially mitigated the SA-β-gal activity induced by cyclosporin A (CsA), the proportion of SA-β-gal-positive cells decreased more significantly after treatment with KL-EVs (Figure 5A). Concordantly, it was substantiated that the mRNA expression levels of *CDKN2A*, *CDKN1A*, and *TP53* (Figure 5B), along with two key senescence-associated secretory phenotype (SASP) components *IL1B* and *IL6*, were all effectively decreased in HK-2-cell-treated KL-EVs (Figure 5C).

Renal fibrosis is one of the terminal manifestations of renal aging, and the epithelial–mesenchymal transition (EMT) of renal tubular epithelial cells is a key process leading to renal fibrosis [27]. Intriguingly, qPCR results showed that following treatment with KL-EVs, the expression levels of fibrosis-related genes, *FN1* and *TGF-β1*, were significantly reduced; conversely, the expression level of the epithelial marker E-cadherin (encoded by *CDH1*) was markedly upregulated (Figure 5D), implying that KL-EVs had the potential to inhibit the pathological process of EMT. Moreover, dysregulation of oxidative stress is another important hallmark of renal aging [28]. We found that reactive oxygen species (ROS) levels were significantly elevated in CsA-induced aged HK-2 cells. Both CTRL-EVs and KL-EVs effectively mitigated intracellular ROS accumulation, with KL-EVs exhibiting a more pronounced reduction (Figure 5E). In parallel, we observed that both types of EVs increased catalase (CAT) levels in senescent HK-2 cells, with the KL-EVs group exhibiting significantly higher CAT levels (Figure 5F). Overall, these findings showcased the superior geroprotective features of KL-EVs against EMT and oxidant stress of senescent HK-2 cells.

### 2.6. KL-EVs and CTRL-EVs Contained Abundant Proteins

**Figure 6:**
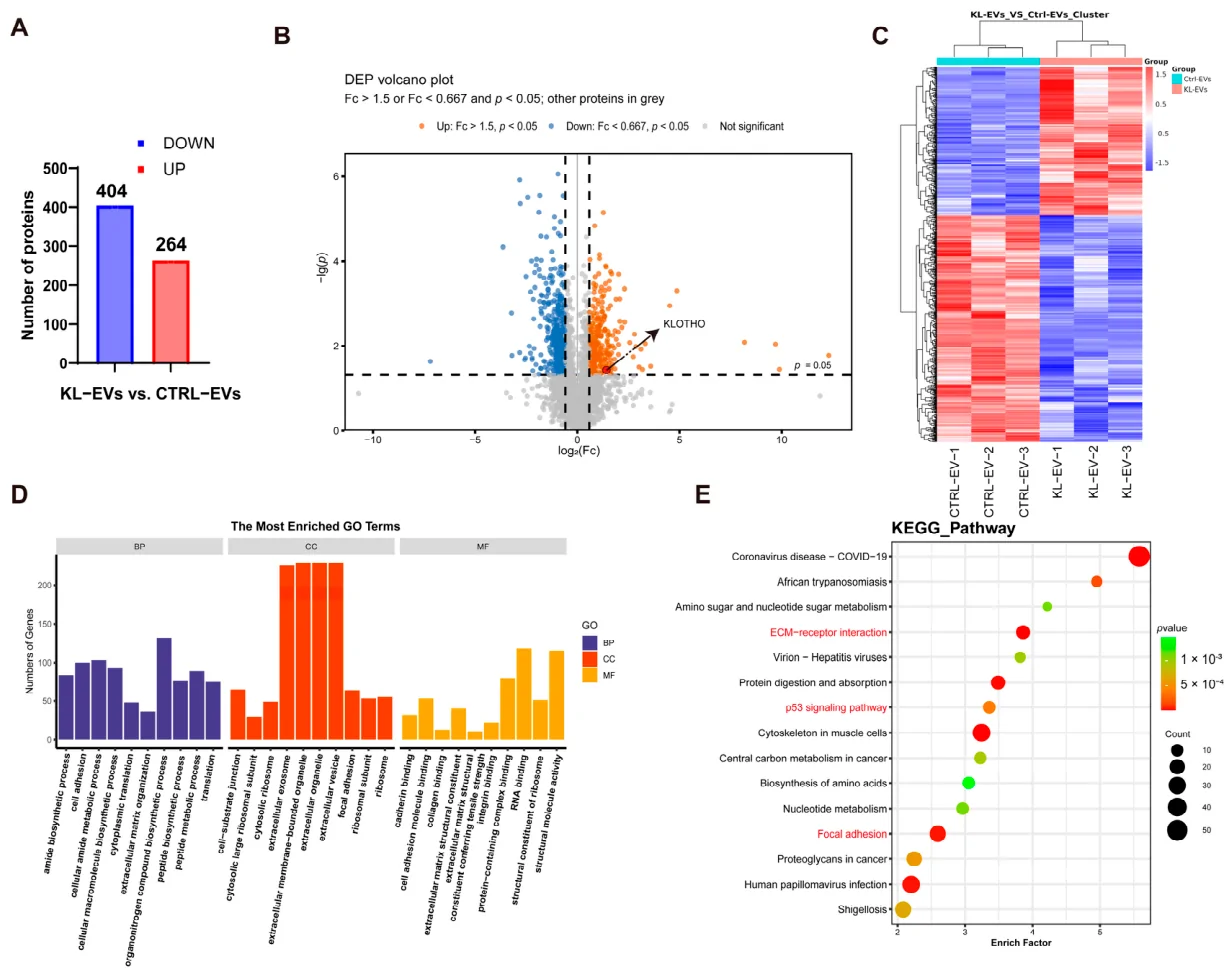
**Proteomic analysis of the cargoes of CTRL-EVs and KL-EVs.** (**A**) Bar graph summarizing differential expressed proteins (DEPs) in CTRL-EV and KL-EV (foldchange, Fc > 1.5 or Fc < 0.667, and *p* < 0.05). (**B**,**C**) Volcano plot and heatmap of the DEPs identified in CTRL-EV and KL-EV. (**D**) Top 10 enriched GO terms with regard to biological process (BP), cell component (CC), and molecular function (MF) for KL-EV highly expressed proteins. (**E**) KEGG pathway classification of upregulated proteins in KL-EVs compared with CTRL-EVs. Proteomic analysis was performed using three independent biological replicates per group (*n* = 3).

We next elucidated the protein compositions of CTRL-EVs and KL-EVs. Our data identified 668 differentially expressed proteins (DEPs) in total (*p <* 0.05). Specifically, 264 proteins showed higher expression and 404 proteins showed lower expression in KL-EVs than CTRL-EVs. SUSD2, SIAE, LFNG, FGD4, AQP1, TMEM168, MEP1A, SCIN, CLDN5, and RDH10 were the top ten up-regulated proteins, whereas EPHB1, C1QTNF1, PAG1, MXRA5, TNC, MX1, SRPX2, CLDN12, CILP, LRRC17 were the top ten downregulated proteins (Figure 6A–C). The up-regulated expression of the Klotho protein in KL-EVs was confirmed (Figure 6B).

Gene ontology (GO) analysis of DEPs further characterized the protein composition of KL-EVs. In the cellular component (CC) category, DEPs were predominantly enriched in extracellular vesicle-, extracellular exosome-, and membrane-bounded organelle-related terms, supporting the vesicular origin and high purity of the isolated EVs. Molecular function (MF) and biological process (BP) analyses showed enrichment in pathways associated with cell adhesion, extracellular matrix organization, and cadherin binding, suggesting a potential role for KL-EVs in regulating cell–matrix interactions and tissue remodeling. Additionally, several terms related to translation, RNA binding, and ribosomal components were significantly enriched, implying that Klotho enrichment may alter the protein cargo profile of EVs and influence cellular processes involved in protein synthesis and homeostasis (Figure 6D).

To further explore the biological pathways associated with these proteomic changes, Kyoto Encyclopedia of Genes and Genomes (KEGG) enrichment analysis was performed. Among the significantly enriched pathways, the p53 signaling pathway was prominently represented, consistent with the suppression of the p53–p21 axis observed in both BMSC and HK-2 senescence models. In parallel, pathways related to ECM–receptor interaction and focal adhesion were also enriched (Figure 6E). Given the established roles of these pathways in stem cell fate regulation and cell–matrix communication, their enrichment may contribute to the tissue repair and regeneration mediated by KL-EV treatment.

## 3. Discussion

The present study was motivated by the growing recognition that engineering UC-MSCs to enrich therapeutic cargo within their EVs may substantially enhance their regenerative efficacy. Among the potential candidates, Klotho is of particular interest because its expression is relatively low in native UC-MSCs and their EVs despite its well-established anti-aging and tissue-protective functions [20,29]. However, the clinical translation of Klotho-based therapies remains challenging owing to the large molecular size of the full-length protein, its limited stability in vivo, and the lack of efficient delivery systems. Although several Klotho-derived peptides have recently been developed to recapitulate specific biological activities of Klotho [30,31,32,33], these peptide-based approaches may not fully reproduce the broad spectrum of functions mediated by the intact protein. As the mounting literature suggests, full-length Klotho is primarily expressed as a transmembrane protein on renal tubular epithelial cells and is inextricably associated with renal aging, fibrosis and the development of various diseases [34,35,36,37]; more importantly, the extracellular domain of Klotho, following cleavage by hydrolases, acts as a soluble hormone to mediate the rejuvenation of multiple organ functions throughout the body [38,39,40]. In order to both reproduce the functional integrity of this longevity factor and ensure the efficiency of genetic engineering, we generated UC-MSCs overexpressing the functional extracellular domain of Klotho (34-981aa) and made it possible to isolate EVs enriched with Klotho. Our strategy combines the potential biological activities of EV-associated Klotho with the intrinsic bioactivity and delivery properties of UC-EVs to ameliorate cellular senescence.

As a key cell source in regenerative medicine, UC-MSCs possess a broad spectrum of biological functions, including multilineage differentiation capacity, pro-angiogenic activity, and immunomodulatory property, making them an appealing candidate for tissue repair and regeneration. Against this background, KL-UC-MSCs exhibited enhanced regenerative properties. The supernatant from KL-UC-MSCs more effectively promoted endothelial cell migration, scratch closure, and tube formation, indicating a salient pro-angiogenic phenotype. These effects may reflect the enhanced stemness of engineered MSCs and the consequent release of as-yet-unidentified regenerative factors, including EVs. More importantly, KL-UC-MSCs displayed more pronounced osteogenic differentiation potential. This finding is consistent with the established role of Klotho in osteogenesis-related signaling pathways [41] and further suggests that UC-MSCs overexpressing Klotho may coordinately regulate the osteogenic–angiogenic coupling required for effective bone regeneration. To our knowledge, how Klotho dictates MSC fate commitment has not been fully reported. Whether Klotho promotes osteogenic differentiation of MSCs through the classic Wnt/β-catenin or BMP/Smad signaling pathways remains to be determined. Although these pathways have been implicated in Klotho-mediated effects in renal diseases, their roles in driving the pro-osteogenic effects in MSCs have yet to be established. Elucidating the signaling networks underlying these effects may therefore provide important mechanistic insights into MSC biology and skeletal aging. On all accounts, our data implied that the novel UC-MSCs represent a promising agent to promote bone repair and remodel aged bone marrow niche.

Inspired by the aforementioned findings that KL-UC-MSCs exhibited a more potent osteogenic differentiation capacity, we intentionally focused on BMSCs as the EV intervention target, whose age-associated shift from osteogenesis toward adipogenesis is recognized as a key contributor to skeletal aging and deterioration of the bone marrow niche. Consistent with our previous findings that native UC-EVs partially restored the phenotypes of aged BMSCs [5], Klotho enrichment further enhanced the ability of EVs to promote osteogenic differentiation. Although both CTRL-EVs and KL-EVs tended to suppress adipogenic differentiation, KL-EVs exhibited superior efficacy in restoring osteogenic potential, suggesting a stronger capacity to counteract age-related functional decline in BMSCs. These findings support the potential application of KL-EVs in skeletal degenerative disorders characterized by impaired bone formation and stem cell dysfunction. Crucially, the beneficial effects of KL-EVs were not restricted to the skeletal system. Given the well-established role of Klotho in renal homeostasis and aging, we further evaluated KL-EVs in a CsA-induced senescent HK-2 cell model. KL-EVs effectively alleviated cellular senescence, suppressed EMT progression, and reduced oxidative stress, indicating a broader anti-aging activity across distinct tissue types. These observations provide preliminary evidence that the Klotho-derived fragment enriched in EVs possesses essentially the same function as the full-length version, even if further investigation is required to fully characterize its effects. Although promising, it is important to note that proteomics results suggest that the cargoes of KL-EVs generated using this method contain many DEPs other than Klotho. Hence, at this stage, it cannot be concluded that the enhanced function of KL-EVs is entirely attributable to the increased enrichment of Klotho, as this may be the result of the combined action of Klotho and other factors. Collectively, Klotho-enriched EVs may function as a versatile regenerative platform capable of targeting multiple aging-associated pathological processes beyond the bone marrow microenvironment.

The successful implementation of such a strategy, however, relied on the efficient sorting of therapeutic cargoes into EVs. In this study, VSVG was selected as a scaffold protein because this virus-related scaffold protein has been reported to facilitate the effective loading of macromolecular cargoes into EVs, ensure cytosolic release and safeguard target proteins against lysosomal degradation [42,43,44,45]. Recent advances in EV engineering have generated a variety of alternative loading platforms, based on tetraspanins, type membrane proteins, peripheral membrane proteins, virus-related proteins and lipid-anchored proteins, each with distinct advantages regarding loading efficiency, targeting capability, and manufacturability [46]. Our findings demonstrated that VSVG-mediated engineering enables effective enrichment of Klotho within EVs while preserving their biological activity. It is noteworthy in the future that potential immunogenicity associated with VSVG may be further mitigated through glycoengineering strategies designed to shield exposed antigenic epitopes and reduce immune recognition [47]. Nevertheless, systematic comparisons among different scaffold proteins remain limited and deciphering the location of Klotho necessitates additional topology evidence. Future studies evaluating cargo-loading efficiency, target molecule localization, in vivo biodistribution, and long-term biosafety across different EV engineering platforms will be critical for optimizing the clinical translation of engineered EV therapeutics.

The main limitation of this study lies in the lack of support from in vivo experimental results. Future research will investigate the optimal dosage and administration routes of CTRL-EVs and KL-EVs in models of natural aging and related disease models, with a focus on observing their effects on the repair of skeletal and renal damage as well as age-related degenerative changes. Further investigation into the safety concerns, including the probability of pulmonary embolism, allergic reactions, or other toxic responses in mice following EV administration, is warranted.

## 4. Materials and Methods

### 4.1. Cell Culture

Human umbilical cord samples were collected from full-term infants delivered via a cesarean section at Wuhan Union Hospital, with prior informed consent obtained from all donors. The use of human umbilical cord tissue was approved by the Ethics Committee of the Union Hospital, Tongji Medical College, Huazhong University of Science and Technology (UHCT-IEC-SOP-016-02-02). UC-MSCs were isolated and characterized as previously described [48]. Human umbilical vein endothelial cells (HUVECs), BMSCs and HK-2 cells were purchased from commercial sources.

Basal culture media were specific to each cell type and supplemented with 10% fetal bovine serum (FBS; NEWZERUM, Christchurch, New Zealand) and 100 U/mL penicillin/streptomycin (Solarbio, Beijing, China). UC-MSCs and HK-2 cells were cultured in Dulbecco’s modified Eagle’s medium (DMEM)/F-12 medium (Gibco, Grand Island, NY, USA). HUVECs were cultured in DMEM (Gibco, Grand Island, NY, USA). BMSCs were cultured in α-MEM (Gibco, Grand Island, NY, USA). To minimize potential confounding from bovine EVs, FBS used for UC-MSCs culture was pre-cleared of endogenous EVs by centrifuge at 20,000× *g* for 90 min at 4 °C before being added to the culture medium. All cell types were maintained in a humidified incubator at 37 °C with 5% CO_2_. Serial passaging was performed when the aforementioned cells reached 80–90% confluence and were still actively dividing. Experimental procedures were conducted using UC-MSCs within passage 10.

### 4.2. Design and Transduction of VSVG-Klotho Lentiviral Vectors

In this study, GV717 (CMV enhancer-MCS-sv40-puro) was employed to contain SP-VSVG-EGFP-Klotho (34-981) (Table S1), and it serves as a negative control per se. These lentiviruses were synthesized by GeneChem Co., Ltd. (Shanghai, China). with HitransG P reagent; the lentivirus was incubated at a multiplicity of infection of 75 in UC-MSCs (seeded at 1 × 10^5^ cells/well in 6-well plates). After 20 h of culture, the viral supernatants were removed, and the cells were washed with PBS. Fresh medium was added, and cells were cultured for an additional 48 h. After the selection via 2 μg/mL Puromycin (Beyotime, Shanghai, China), the transduced cells were cultured in the same manner as that used for the UC-MSCs.

### 4.3. Generation and Isolation of EVs

According to previous publications [5,48], the supernatants of UC-MSCs were collected for EV isolation. The culture medium underwent a series of centrifugation steps to isolate the EVs. Initially, the medium was centrifuged at 750× *g* for 15 min to eliminate cells, followed by centrifugation at 2000× *g* for 20 min to remove apoptotic bodies and debris. Subsequently, the supernatants were subjected to further centrifugation at 18,000× *g* (Avanti J-26S XPI High-Performance Centrifuge; Beckman Coulter, Brea, CA, USA) for one hour at 4 °C to pelletize the EVs. After washing and resuspension in sterile PBS, the EVs were either used immediately or stored frozen at −80 °C until needed. The number of CTRL-EVs and KL-EVs obtained was determined by lysing purified EV particles in RIPA buffer and quantifying the total protein content via a BCA protein assay.

### 4.4. Transmission Electron Microscopy

Briefly, 10 μL CTRL-EVs and KL-EVs were deposited onto a 200-mesh copper grid for a duration of 2 min and gently dried by blotting with filter paper. Next, the grids were stained with 10 μL of 2% uranyl acetate in the absence of light for a period of 3 min. Finally, the grids were visualized via the microscopy (Model HT7800, Hitachi, Tokyo, Japan) at 80.0 kV.

### 4.5. Nanoparticles Tracking Analysis

The size distribution and concentration of CTRL-EVs and KL-EVs were characterized by nanoparticle tracking analysis using a ZetaView platform (Particle Metrix, Meerbusch, Germany). CTRL-EVs and KL-EVs were appropriately diluted with PBS and analyzed at room temperature. The scattered signals were amplified 10-fold to visualize the EV particles and track their Brownian motion.

### 4.6. Zeta Potential Detection

Dilute the CTRL-EVs and KL-EVs suspensions to a suitable concentration, dispense 1 mL of the sample into the sample cell of the ZetaView analyzer (Particle Metrix, Meerbusch, Germany), and record and calculate the Zeta potential values at different positions.

### 4.7. Enzyme-Linked Immunosorbent Assay (ELISA)

The Klotho content in the lysate of KL-EVs and CTRL-EVs was quantified using the Human KL/Klotho ELISA Kit (RK04743, ABclonal, Wuhan, China), following the protocol provided by the manufacturer.

### 4.8. EV Treatment In Vitro

Initially, HK-2 cells or adult BMSCs were seeded at 1 × 10^5^ cells/well in 6-well plates. HK-2 cells were stimulated with cyclosporin A (CsA, 30 μM, MedChemExpress, Monmouth Junction, NJ, USA) for 2 days to establish a senescent renal tubular epithelial cell model [49]. After the cells were washed with PBS, fresh medium was added. The cells were separately treated with CTRL-EVs (10 μg/mL), KL-EVs (10 μg/mL), or an equal volume of PBS (as a control) once a day. After 2 days, the cells were collected for further analysis.

### 4.9. Flow Cytometry

Due to the autofluorescence of GFP, the transduced cells were collected and analyzed directly to determine the percentage of GFP-positive cells. For characterization, UC-MSC suspension was incubated with anti-human CD29, CD44, CD90, CD105, CD34, and CD45 antibodies for 30 min at 4 °C before analysis with a FACS Calibur (BD Biosciences, San Jose, CA, USA).

### 4.10. RNA Isolation and Quantitative Real-Time PCR

The RNA Easy Isolation Reagent (Vazyme, Nanjing, China) was used to extract total RNA from cells. HiScript III RT Supermix for qPCR (Vazyme) was employed for the reverse transcription of RNA samples. Template cDNA was then subjected to real-time polymerase chain reaction using ChamQ SYBR qPCR Master Mix (Vazyme) The StepOnePlus Real-Time PCR System (Thermo Fisher Scientific, Inc., Waltham, MA, USA) was utilized for qPCR reaction. Relative gene expression levels were calculated using the 2^−ΔΔCt^ method and normalized to the corresponding reference gene. The primer sequences are listed in Table S2.

### 4.11. Western Blotting

Cells or EVs were subjected to lysis in ice-cold RIPA buffer supplemented with phenylmethyl sulfonyl fluoride and phosphatase inhibitors (Beyotime, Shanghai, China) at 4 °C for 30 min. Proteins were quantified using the BCA assay, denatured, separated on SDS-polyacrylamide gels (EpiZyme, Cambridge, MA, USA) and then transferred onto PVDF membranes (Merck, Rahway, NJ, USA; Millipore, Burlington, MA, USA). For immunoblotting, PVDF membranes were blocked in Fast Blocking Buffer (Servicebio, Wuhan, China) for 30 min at room temperature and proceeded to incubate with primary antibodies at 4 °C overnight. On the second day, the PVDF membranes were washed with 1 × TBST solution and proceeded to incubate with HRP-conjugated secondary antibodies for 1 h at room temperature. PVDF membranes were washed with 1 × TBST solution and analyzed using a ChemiScope 6200 imaging system (Clinx Sciecne Instruments Co., Ltd., Shanghai, China). The antibody information is displayed in Table S3.

### 4.12. Scratch Wound Assay

Conditioned media (CM) of UC-MSCs were harvested and centrifuged at 1000× *g* for 20 min to remove cell debris. HUVECs were seeded at 1 × 10^5^ cells/well in 6-well plates. When HUVECs reached confluence, cells were treated with mitomycin C for 2 h at 37 °C. The cells were washed with PBS and scratched using a p200 pipet tip. After culturing in CM from UC-MSCs for 0, 12, and 24 h, scratch wounds were photographed under a phase-contrast microscope (Olympus). The cell migration rate was calculated as follows: migration area (%) = (A_0_ − A_n_)/A_0_ × 100%, where A_0_ is the initial wound area and A_n_ is the remaining wound area at the indicated time point.

### 4.13. Tube Formation Assay

A total of 2 × 10^4^ HUVECs were seeded on pre-cooled Matrigel (BD Biosciences, San Jose, CA, USA)-coated wells of a 96-well plate and cultured in CM from UC-MSCs. After incubating the cells for 4 h, tube formation was photographed under an inverted microscope (Olympus Corporation, Tokyo, Japan). The number of meshes was analyzed using ImageJ software (v1.54).

### 4.14. 5-Ethynyl-2′-Deoxyuridine (EdU) Staining

DNA synthesis was detected using a BeyoClick EdU-594 Cell Proliferation Kit (Beyotime, Shanghai, China) in accordance with the manufacturer’s instructions. After treating the cells with EVs, incubate them in a 10 μM EdU working solution in the incubator for 10 h. On labeling, fix the cells with 4% paraformaldehyde at room temperature for 15 min and wash with PBS, followed by 15 min of incubation with permeabilization solution at room temperature. After washing twice, a fresh Click reaction mixture was added and then incubated at room temperature in the dark for 30 min. Hoechst 33342 was employed for nuclear staining. Cells were observed under a fluorescence microscope.

### 4.15. Senescence-Associated β-Galactosidase (SA-β-Gal) Staining Assay

After treatment, BMSCs and HK-2 cells were fixed in fixative solution for 15 min, stained with SA-β-gal staining solution (Beyotime, Shanghai, China), and incubated overnight at 37 °C. The SA-β-gal-positive cells were visualized with a microscope (Olympus Corporation, Tokyo, Japan) and quantified with ImageJ software.

### 4.16. Osteogenic Differentiation

Induction of differentiation: UC-MSCs and BMSCs were cultured in 6-well plates in standard complete medium until the cell density reached 100%, then switched to osteogenic induction medium prepared in advance according to the manufacturer’s instructions (Cyagen, Santa Clara, CA, USA). A complete medium change was performed every 3 days and the induction was continued for 14 days.

Alizarin red staining: Aspirate the medium, wash twice with PBS, and fix with 4% paraformaldehyde at room temperature for 30 min. Add Alizarin red working solution, and stain at room temperature for 10 min. After washing twice with PBS, images were obtained under a microscope.

Alkaline phosphatase (ALP) staining: Remove the culture medium, wash once with PBS, fix for 1 min using ALP fixative pre-chilled to 4 °C, wash once with distilled water, add freshly prepared ALP incubation solution (Solarbio, Beijing, China) to cover the cells, incubate in a cell culture incubator for 20 min, wash once with distilled water, stain with methyl green counterstain for 5 min, wash, and observe under a microscope.

### 4.17. Adipogenic Differentiation

UC-MSCs and BMSCs were cultured in 6-well plates in standard complete medium until the cell density reached 100%, then switched to adipogenic induction medium prepared in advance according to the manufacturer’s instructions (Cyagen, Santa Clara, CA, USA). A complete medium change was performed every 3 days and the induction was continued for 14 days The cells were stained with an Oil Red O solution (Beyotime, Shanghai, China) to distinguish mature adipocytes from preadipocytes.

### 4.18. Reactive Oxygen Species (ROS) Detection

Following the kit instructions (Beyotime, Shanghai, China), HK-2 cells were seeded in a 6-well plate. The cells were first treated with cyclosporine A and EVs, followed by in situ probe loading, which involved adding 1 mL of DCFH-DA diluted 1:2000, and incubated in a cell culture incubator for 20 min. After washing the cells three times with PBS, observation was performed using an inverted fluorescence microscope.

### 4.19. Catalase Assay

Collect cells from each group, sonicate them in an ice-water bath, centrifuge at 4000 rpm for 10 min, and collect the supernatant. Determine the protein concentration using the BCA method. Add reagents in sequence according to the kit instructions (A007-1-1, from Nanjing Jiancheng Bioengineering Institution, Nanjing, China), incubate at 37 °C for exactly 1 min, and dispense 200 μL of the reaction mixture into a 96-well plate, read the absorbance at 405 nm using a microplate reader, and calculate ΔA = A (control) − A (sample). Calculate the catalase content for each group using the established formula.

### 4.20. Proteomic Analysis of CTRL-EVs and KL-EVs

EV proteomics sequencing and analysis were conducted in collaboration with Wayen Biotechnologies, Inc, Shanghai, China. After extraction, proteolysis and desalting of the EV proteins, the samples were identified by LC-MS/MS. For library preparation, proteins extracted from each sample after removal of high-abundance proteins using the High Select™ Top 14 Abundant Protein Depletion Midi Spin Columns kit (Thermo Fisher Scientific, Waltham, MA, USA, A36371) were mixed in equal volumes. The mixture was fractionated using high-pH ultra-high-performance liquid chromatography, followed by data acquisition via the DDA method for library construction. Subsequently, DIA data acquisition was performed on each sample in sequence for relative quantification analysis. The acquired data were normalized and used for relative protein quantification using Spectronaut 11.0 (Biognosys AG, 2013) software. The screening criteria for differentially expressed proteins were set at *p* < 0.05 and foldchange > 1.5 or <0.667.

### 4.21. Statistical Analysis

One-way analysis of variance (ANOVA), two-way ANOVA, and Student’s *t*-test were performed utilizing GraphPad Prism 10.0 software (GraphPad software, La Jolla, CA, USA) to analyze the data. All the data are presented as the Mean ± Standard Derivation. *p* < 0.05 was considered to indicate statistical significance.

## 5. Conclusions

In this study, a truncated fragment containing the functional domain of Klotho was selected and fused with the scaffold protein VSVG for co-expression. Through lentiviral transduction of UC-MSCs, we generated a novel type of UC-MSCs that highly expresses Klotho and isolated Klotho-rich EVs released by these cells. The novel UC-MSCs overexpressing Klotho retained their inherent morphological and immunophenotypic characteristics while exhibiting significantly bolstered angiogenic potential and osteogenic differentiation capacity. Furthermore, the Klotho-enriched UC-EVs effectively improve various age-related phenotypes of adult BMSCs and aged HK-2 cells, providing solid evidence for their comprehensive anti-aging efficacy.

## Data Availability

The datasets generated and analyzed during the current study are available from the corresponding author upon reasonable request.

## Supplementary Materials

The following supporting information can be downloaded at: https://www.mdpi.com/article/10.3390/ijms27188326/s1.
