# Supplementary material for: Characterization of Umbilical Cord Mesenchymal Stromal Cells Overexpressing Klotho and In Vitro Anti-Aging Efficacy of Their Derived Extracellular Vesicles
Source: Int J Mol Sci. 2026 Sep 19;27(18):8326. doi: 10.3390/ijms27188326 (PMC13607764; doi:10.3390/ijms27188326)
Supplement: Supplementary file 1 [file ijms-27-08326-s001.zip › ijms-4545972-supplementary.pdf]

Table S1 Sequence of the VSVG-EGFP-Klotho fusion construct

SP-VSVG-EGFP-KLOTHO(34-981aa)
MFMPSSLSYSSWATCWLLCCLIILAKNSSTDPSRNSDTMKCLLYLAFLFIGVNCKFTIVFPHNQKG
NWKNVPSNYHYCPSSSDLNWHNDLIGTALQVKMPKSHKAIQADGWMCHASKWVTTCDFRWY
GPKYITHSIRSFTPSVEQCKESIEQTKQGTWLNPGFPPQSCGYATVTDAEAVIVQVTPHHVLVDEY
TGEWVDSQFINGKCSNYICPTVHNSTTWHSDYKVKGLCDSNLISMDITFFSEDGELSSLGKEGTGF
RSNYFAYETGGKACKMQYCKHWGVRLPSGVWFEMADKDLFAAARFPECPEGSSISAPSQTSVD
VSLIQDVERILDYSLCQETWSKIRAGLPISPVDLSYLAPKNPGTGPAFTIINGTLKYFETRYIRVDIA
APILSRMVGMISGTTTERELWDDWAPYEDVEIGPNGVLRTSSGYKFPLYMIGHGMLDSDLHLSSK
AQVFEHPHIQDAASQLPDDESLFFGDTGLSKNPIELVEGWFSSWKSSIASFFFIIGLIIGLFLVLRVGI
HLCIKLKHTKKRQIYTDIEMNRLGKMVSKGEELFTGVVPILVELDGDVNGHKFSVSGEGEGDAT
YGKLTLKFICTTGKLPVPWPTLVTTLTYGVQCFSRYPDHMKQHDFFKSAMPEGYVQERTIFFKDD
GNYKTRAEVKFEGDTLVNRIELKGIDFKEDGNILGHKLEYNYNSHNVYIMADKQKNGIKVNFKI
RHNIEDGSVQLADHYQQNTPIGDGPVLLPDNHYLSTQSALSKDPNEKRDHMVLLEFVTAAGITLG
MDELYKEPGDGAQTWARFSRPPAPEAAGLFQGTFPDGFLWAVGSAAYQTEGGWQQHGKGASI
WDTFTHHPLAPPGDSRNASLPLGAPSPLQPATGDVASDSYNNVFRDTEALRELGVTHYRFSISWA
RVLPNGSAGVPNREGLRYYRRLLERLRELGVQPVVTLYHWDLPQRLQDAYGGWANRALADHF
RDYAELCFRHFGGQVKYWITIDNPYVVAWHGYATGRLAPGIRGSPRLGYLVAHNLLLAHAKVW
HLYNTSFRPTQGGQVSIALSSHWINPRRMTDHSIKECQKSLDFVLGWFAKPVFIDGDYPESMKNN
LSSILPDFTESEKKFIKGTADFFALCFGPTLSFQLLDPHMKFRQLESPNLRQLLSWIDLEFNHPQIFI
VENGWFVSGTTKRDDAKYMYYLKKFIMETLKAIKLDGVDVIGYTAWSLMDGFEWHRGYSIRRG
LFYVDFLSQDKMLLPKSSALFYQKLIEKNGFPPLPENQPLEGTFPCDFAWGVVDNYIQVDTTLSQ
FTDLNVYLWDVHHSKRLIKVDGVVTKKRKSYCVDFAAIQPQIALLQEMHVTHFRFSLDWALILP
LGNQSQVNHTILQYYRCMASELVRVNITPVVALWQPMAPNQGLPRLLARQGAWENPYTALAFA
EYARLCFQELGHHVKLWITMNEPYTRNMTYSAGHNLLKAHALAWHVYNEKFRHAQNGKISIAL
QADWIEPACPFSQKDKEVAERVLEFDIGWLAEPIFGSGDYPWVMRDWLNQRNNFLLPYFTEDEK
KLIQGTFDFLALSHYTTILVDSEKEDPIKYNDYLEVQEMTDITWLNSPSQVAVVPWGLRKVLNWL
KFKYGDLPMYIISNGIDDGLHAEDDQLRVYYMQNYINEALKAHILDGINLCGYFAYSFNDRTAPR
FGLYRYAADQFEPKASMKHYRKIIDSNGFPGPETLERFCPEEFTVCTECSFFHTRKS

Table S2 Primers for qPCR

Gene	Primer Forward	Primer Reverse
CDKN2A	5’-CCGTGGACCTGGCTGAGGAG-3’	5’-CGGGGATGTCTGAGGGACCTTC-3’
CDKN1A	5’-GCCCGTGAGCGATGGAACTTC-3’	5’-CCTGCCTCCTCCCAACTCATCC-3’
TP53	5’-GAGGTTGGCTCTGACTGTACC-3’	5’-TCCGTCCCAGTAGATTACCAC-3’
IL1B	5’-TCCGACCACCACTACAGCAAGG-3’	5’-GGGCAGGGAACCAGCATCTTC-3’
IL6	5’-GGTGTTGCCTGCTGCCTTCC-3’	5’-TGAGATGCCGTCGAGGATGTACC-3’
Klotho	5’-GGATAGAACCTGCCTGCCCTTTC-3’	5’-CTCAGCCAGCCAGCCAATGTC-3’
EGFP	5’-CTTCAAGGACGACGGCAACTAC-3’	5’-CTTCAGCTCGATGCGGTTCAC-3’
RUNX2	5’-AATGGTTAATCTCCGCAGGTC-3’	5’-TTCAGATAGAACTTGTACCCTCTGTT-3’
BMP2	5’-CCAGCCGAGCCAACACTGTGC-3’	5’-TCTCCGGGTTGTTTTCCCACTCG-3’
BGLAP	5’-AGCAAAGGTGCAGCCTTTGT-3’	5’-GCGCCTGGGTCTCTTCACT-3’
ALPL	5’-AACACCACCCAGGGGAAC-3’	5’-TGGCATGGTTCACTCTCGT-3’
PPARG	5’-TGCAGTGGGGATGTCTCATA-3’	5’-CAGCTGGTCGATATCACTGGA-3’
FABP4	5’-GGCCAGGAATTTGACGAAGT-3’	5’-TTTCCATCCCATTTCTGCAC-3’
LPL	5’-CCCTAAGGACCCCTGAAGAC-3’	5’-GGTTTTGCTGCTGTGATTGA-3’
NANOG	5’-CTGCAGAGAAGAGTGTCGCA-3’	5’-ACATTAAGGCCTTCCCCAGC-3’
OCT4	5’-TCAAAGCAAGCTGGGGAGAG-3’	5’-ACATCCAAGGGATGCAGAGC-3’
FN	5’-CGGTGGCTGTCAGTCAAAG-3’	5’-AAACCTCGGCTTCCTCCATAA-3’
TGFB1	5’-GCAACAATTCCTGGCGATACCTC-3’	5’-CCTCCACGGCTCAACCACTG-3’
ACTB	5’-CAGGGCGTGATGGTGGGCA-3’	5’-CAAACATGATCTGGGTCATCTTCT-3’
GAPDH	5’-CTCTGCTCCTCCTGTTCGACA-3’	5’-ACGACCAAATCCGTTGACTC-3’

Table S3 Antibodies used for flow cytometry and Western blotting

**Antibodies**	**Source**	**Identifier**
Flow cytometry		
Anti-CD29	BD Biosciences	Cat# 556049
Anti-CD34	BD Biosciences	Cat# 555822
Anti-CD44	BioLegend	Cat# 103203
Anti-CD45	BD Biosciences	Cat# 555483
Anti-CD90	BD Biosciences	Cat# 555596
Anti-CD105	ABclonal	Cat# A24319
IgG2b κ Isotype Control	BD Biosciences	Cat# 556078
Western blotting		
Anti-CD9	ABclonal	Cat# A19027
Anti-CD63	Bioss	Cat# bs-1523R
Anti-CD81	ABclonal	Cat# A22528
Anti-TSG101	Proteintech	Cat# 28283-1-AP
Anti-P21	Cell Signaling Technology	Cat# 2947T
Anti-KLOTHO	Proteintech	Cat# 28100-1-AP
Anti-actin	Abcam	Cat# ab8226
Anti-GAPDH	Abcam	Cat# ab8245
